# Recent Progress in Drug Release Testing Methods of Biopolymeric Particulate System

**DOI:** 10.3390/pharmaceutics13081313

**Published:** 2021-08-23

**Authors:** Yejin Kim, Eun Ji Park, Tae Wan Kim, Dong Hee Na

**Affiliations:** 1 College of Pharmacy, Chung-Ang University, Seoul 06974, Korea; yejin.kim@g2gbio.com (Y.K.); ezkimtaewan@naver.com (T.W.K.); 2 G2GBIO, Inc., Daejeon 34054, Korea; 3 D&D Pharmatech, Seongnam 13486, Korea; ejpark@ddpharmatech.com

**Keywords:** biopolymeric microparticles, poly(lactide-co-glycolide), drug release testing, in vitro–in vivo correlation, accelerated in vitro release testing methods

## Abstract

Biopolymeric microparticles have been widely used for long-term release formulations of short half-life chemicals or synthetic peptides. Characterization of the drug release from microparticles is important to ensure product quality and desired pharmacological effect. However, there is no official method for long-term release parenteral dosage forms. Much work has been done to develop methods for in vitro drug release testing, generally grouped into three major categories: sample and separate, dialysis membrane, and continuous flow (flow-through cell) methods. In vitro drug release testing also plays an important role in providing insight into the in vivo performance of a product. In vitro release test with in vivo relevance can reduce the cost of conducting in vivo studies and accelerate drug product development. Therefore, investigation of the in vitro–in vivo correlation (IVIVC) is increasingly becoming an essential part of particulate formulation development. This review summarizes the principles of the in vitro release testing methods of biopolymeric particulate system with the recent research articles and discusses their characteristics including IVIVC, accelerated release testing methods, and stability of encapsulated drugs.

## 1. Introduction

Biopolymer-based microparticles have been employed for the controlled release of small organic molecules, peptides, and proteins in the various routes including subcutaneous or intramuscular injection and pulmonary inhalation [1,2,3]. Particularly, microparticles made of biodegradable polymers such as poly(lactide) (PLA) and poly(lactide-co-glycolide) (PLGA) are preferred owing to their biocompatible properties [4,5]. Biodegradable microparticle-based drug delivery system provides several advantages over conventional dosage forms such as tablets, capsules, and direct injection of free drugs intravenously or subcutaneously, because they can protect drug molecules from chemical and enzymatic degradation and provide long-acting formulations through continuous and controlled drug release [6]. The advantage of PLA- or PLGA-based microparticles is more distinct in the injectable formulations of drugs with short circulation half-life, because the microparticles could provide reduced frequency of administration through long-term drug release, enhanced stability, and increased patient compliance [7,8,9]. Using PLA/PLGA-based microparticles, more than 15 drugs have been approved by the US Food and Drug Administration (FDA) or European Medicines Agency (EMA), as shown in Table 1 [2,10].

Drug release kinetics from biodegradable microparticles are controlled by diffusion, erosion or a combination of both, typically featuring a triphasic profile: (i) an initial burst release of drugs at or near the polymer surface, (ii) a lag phase during erosion process of polymer, (iii) a secondary release on bulk erosion of the polymer with zero order release kinetics [11]. While the initial burst release is controlled by only diffusion, the lag phase and secondary release are dependent on both diffusion and particle erosion [4]. After administration of microparticle preparations, water absorption by PLA or PLGA polymers in microparticles creates pores inside the polymer matrix as a function of time and leads to a porous interconnected network that allows diffusion of drug molecules from the polymer matrix [12]. Hydrolysis through cleavage of the ester backbone of PLA or PLGA polymers leads to polymer degradation, resulting in mass loss of microparticles. For an ideal continuous drug release, the diffusion and erosion processes need to be complementary so that the drug continues to diffuse out of the microparticles [13].

Characterization of the drug release of microparticles products is required to ensure product quality and the desired pharmacological effect. As dissolution test is used to test the quality of orally administered solid drug products, there is a need for an in vitro release test method to control the product quality of biopolymeric microparticles [14]. However, despite the widespread use of microparticles including more than 15 commercially available PLA/PLGA microparticle products, there is no official method for long-term release parenteral dosage forms [15]. Nonetheless, much of the work towards developing methods for in vitro drug release testing has been usually carried out with one or more of the following aims: (i) to predict the drug availability in preliminary stages of product development, (ii) to meet batch specifications, (iii) to assess formulation factors and manufacturing methods of dosage form, (iv) to support labeling claims of the product, and (v) to meet a compendial standards and regulatory requirement [15,16,17]. In addition, in vitro drug release testing plays an important role in providing insight into the product’s in vivo performance [11]. In vitro release tests with in vivo relevance are preferred because they can reduce the cost of conducting in vivo studies and accelerate drug product development [15]. Therefore, investigation of the in vitro–in vivo correlation (IVIVC) is increasingly becoming an essential part of microparticle formulation development [18,19,20,21,22,23].

In this review, we provide an up-to-date review of the in vitro drug release testing methods of long-acting release particulate formulations along with recent studies. The principles of the in vitro release testing methods are summarized and their characteristics including advantages, disadvantages, and applications are discussed with the recent research articles. Then, the development of accelerated in vitro drug release testing, IVIVC and the prospects of these testing methods are discussed.

## 2. In Vitro Drug Release Testing Methods

For oral dosage forms, the “dissolution” test is generally referred to as a test to evaluate product performance because the drug is intended to dissolve rapidly in the test medium, whereas for non-oral dosage forms such as injectable microparticles formulation including transdermal delivery systems and suppositories, the test is preferably referred to as a “drug release” or “in vitro release” test [14]. In vitro drug release testing methods for injectable microparticles formulation have been generally grouped into three major categories: sample and separate, dialysis membrane, and continuous flow techniques [16,17].

### 2.1. Sample and Separate (SS) Method

**Figure 1:**
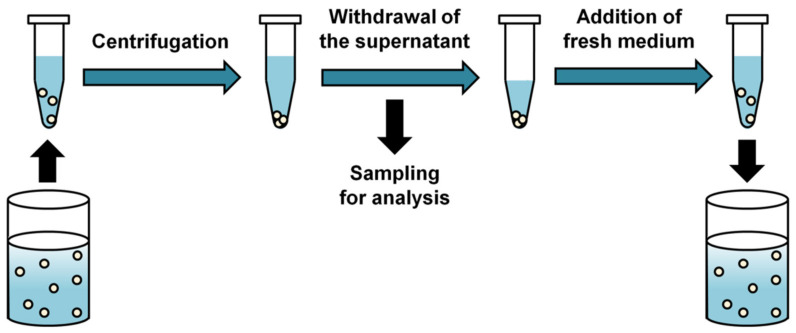
Basic principle and process of sample and separate method for in vitro drug release testing procedure.

The SS method is the simplest and most commonly used for in vitro drug release testing of polymeric microparticle formulations because it provides an easy experimental setup (Figure 1). Typically, drug-encapsulated microparticles are suspended in a container containing a release medium, and then the drug release is measured over time.

In the SS method, the drug release behavior is affected by parameters such as container size, agitation type, sample separation technique, and sampling volume. Containers are usually chosen based on the volume of release media required to retain sink conditions [24]. Generally, tubes or vials were used for small volumes of medium less than 10–15 mL [25,26] and vessels, bottles, or Erlenmeyer flasks were used for larger volumes [27,28]. Agitation of the release medium is an important factor in the in vitro release process, as it can improve particle wetting and accelerate polymer degradation and mass loss [17]. The agitation can be performed continuously or intermittently during the release study. In an in vitro release study with leuprolide-loaded PLGA microparticles, the leuprolide release under continuous agitation was higher than that released under once-a-week agitation [29]. In some cases, the release medium remained static during the release study [30,31].

To monitor the amount of drug released from the microparticles, a sample for analysis is prepared by separating the supernatant from the precipitated microparticles after centrifugation. When the drug is stable in the release medium, the supernatant is periodically taken and the filtered samples are analyzed by spectrophotometric or chromatographic methods [32,33]. For drugs that are unstable in the release medium, residual microparticles are recovered and the drug remaining in the microparticles is analyzed to indirectly determine the amount of drug released [34,35]. In the release study with amoxicillin degraded rapidly in phosphate-buffered saline used as the release medium, the mass balance between the released drug, the drug remaining in the microparticles and the initial drug loading was achieved only when drug degradation in release medium was accounted for [35]. However, monitoring drug release using analysis of the drug remaining in the microparticles is not an attractive option because it requires more complex sample preparation procedure and large amounts of microparticles [16].

After sampling, buffer replacement is required to add an equal volume of fresh medium to the release medium in order to maintain the total volume and sink conditions for the duration of the in vitro release study [31]. In some cases, complete buffer replacement is required to prevent accumulation of drug degradation products in the release medium [36]. After buffer replacement, the centrifuged microparticles are resuspended.

The SS method provides accurate data of the drug release profile in a simple experimental setup, and is particularly useful for measuring initial burst release [30]. However, there are some disadvantages such as disruption of the particles due to centrifugal forces, withdrawal of unwanted microparticles from the release medium, and underestimation of drug release due to particulate aggregation [16,17]. Addition of surfactants such as polyvinyl alcohol (PVA) to the release medium and intermittent shaking of the contents of the medium could be useful to minimize aggregate formation [37].

Various types of SS method under different conditions have been applied to assess in vitro drug release. Guo et al. developed a PLGA microparticles containing donepezil for treatment of Alzheimer’s disease over long periods [38]. In this study, an in vitro release study was performed by suspending approximately 2 mg of microparticles in 6 mL of phosphate-buffered saline (pH 7.4) in a round bottomed 10 mL capped glass centrifuge tube and incubating at 37 °C under horizontal shaking 60 rpm. At each predetermined sampling point, the microparticle sample was centrifuged and the supernatant was separated for drug release analysis. In the meantime, the same volume of fresh medium was added, resuspended, and incubated again. The donepezil-loaded microparticles exhibited a continuous release of about 88% over 10 days without apparent initial burst release.

Park et al. evaluated the in vitro release of a three-month leuprolide acetate depot formulation prepared by spray-drying glacial acetic acid solution of leuprolide acetate and PLA, and freeze-drying in a *d*-mannitol solution [39]. This study used 15-mL conical tube for in vitro release study and the samples were shaken at 25 rpm on a rotary shaker for 84 days at 37 °C. The in vitro release of leuprolide from the microparticles showed approximately 13% of initial burst release on day 1 followed by a sustained release for over 84 days, and the release profile was similar to that of Abbott’s Lucrin Depot.

Gu et al. prepared dexamethasone-loaded PLGA microparticles embedded in different PVA hydrogel coatings and optimized the microparticle composition according to the in vitro drug release profile [40]. For the in vitro release test of the PLGA microparticle/PVA hydrogel composites, approximately 5 mg of the composite samples were immersed in 5 mL of phosphate-buffered saline (pH 7.4) containing 0.1% sodium azide and incubated at 37 °C under constant agitation. At predetermined points, all release media was removed and replenished with an equal volume of fresh media. The collected samples were filtered through a 0.45 μm syringe filter and the dexamethasone concentration in each sample was determined by high-performance liquid chromatography (HPLC) analysis. The optimized composite showed in vitro drug release for more than seven months.

Li et al. prepared regorafenib-loaded PLGA microparticles designed to improve transarterial chemoembolization therapy for hepatocellular carcinoma [41]. For the in vitro release test, an automatic dissolution tester (SOTAX AT 7 smart On-Line System, SOTAX AG, Aesch, Switzerland) was applied and a paddle method was used with stirring speed at 100 rpm. Approximately 10 mg of microparticles were immersed in 800 mL of phosphate buffer (pH 7.4) containing 2% sodium dodecyl sulfate and incubated at 37 °C. For 30 days, 5 mL of sample was withdrawn at predetermined time intervals and an equal volume of fresh medium was added. After filtration through a 0.22 μm syringe filter, the drug concentration in the filtrate was determined by HPLC analysis. The regorafenib-loaded PLGA microparticles exhibited a biphasic release pattern characterized by an initial burst release in the first phase and a sustained release in the second phase.

### 2.2. Dialysis Membrane (DM) Method

The DM method is a widely used and versatile method for testing in vitro drug release of particulate formulations including microspheres [42], nanoparticles [43], and liposomes [44,45]. This method utilizes an appropriate dialysis membrane with a specific molecular-weight cut-off (MWCO) to physically separate the released drug molecules from microparticles by allowing the drug to pass easily through the membrane into the release medium. Drug release is usually assessed with samples taken from the external solution outside the dialysis membrane over time. Compared to the SS method, this method eliminates the need to separate the released compounds from microparticles, making sampling relatively easy and eliminating unwanted microparticle loss during sample preparation and handling [17]. However, slow equilibration with the outer medium limits an accurate measurement of initial drug levels [46]. In addition, the disadvantages of the DM method include the difficulty of achieving adequate agitation to prevent microparticle aggregation within the dialysis bag, the inability to use the drugs that bind to the polymer or the dialysis membrane, and the violation of sink conditions within the dialysis bag [15,16,17].

The DM method has been performed in a clipped bag of the dialysis tubing or in various types of dialyzers [42,47]. Dialysis tubing is an economical tool that is clear, flexible and durable, but this tubing has concerns about handling, closing and sample recovery [16]. Dialyzers are designed for a specific sample volume and are convenient and easy to use [48]. The most common used dialyzers are Float-A-Lyzer (Spectrum Laboratories, Rancho Dominquez, CA, USA), Slide-A-Lyzer (Thermo Scientific, Rockford, IL, USA), Pur-A-lyzer (Sigma-Aldrich, St. Louis, USA), D-Tube (Merck-Millipore, Billerica, MA, USA), and GeBA-flex dialysis tube (Gene Bio-Application Ltd., Kfar Hanagide, Israel).

**Figure 2:**
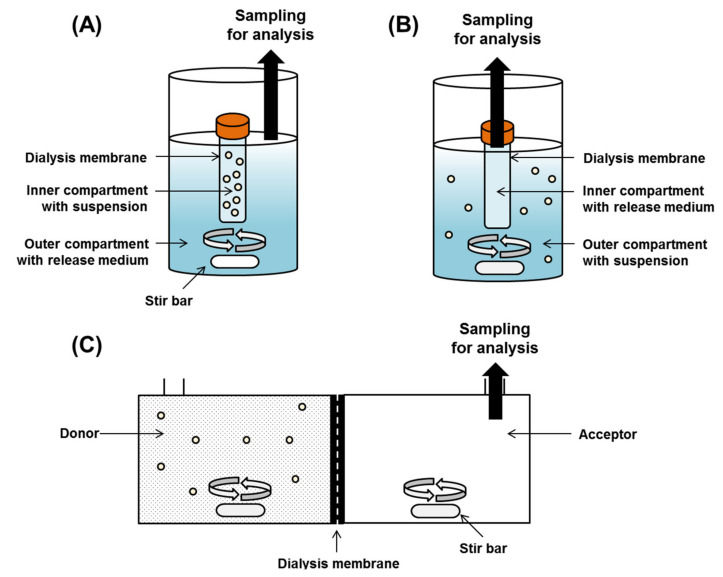
Dialysis membrane methods for in vitro drug release test of particulate formulations: regular dialysis (**A**), reverse dialysis (**B**), and side-by-side dialysis (**C**).

Based on experimental setting, the DM method is classified into regular dialysis, reverse dialysis, and side-by-side dialysis method (Figure 2). In the regular dialysis, the microparticles enter the inside of a sealed dialysis tubing, and drugs released from the microparticles diffuse from the inner medium to the to the outer medium through the dialysis membrane. The diffusion of the drug through the dialysis membrane into the outer medium can be affected by stirring the contents of the container, thus minimizing the effect of the unagitated water layer [16]. Commonly used modes of agitation include a shaker [49,50], magnetic stirrer [51,52], and the United States Pharmacopeia (USP) paddle apparatus under agitation [53]. Unlike regular dialysis, reverse dialysis is a method where the microparticles are placed outside the dialysis tubing and sampling is performed inside the dialysis tubing containing only the medium [54,55]. Sampling is performed by opening the dialysis tubing and removing a certain amount of medium, or by removing the entire dialysis tubing and replacing it with a new one. The main advantage of the reverse dialysis method is that it can avoid the violation of sink condition that occurs in regular dialysis method. In regular dialysis, when the volume inside the dialysis tubing is low and the membrane surface area is small, the rapid drug diffusion of the drug into the bulk release medium of the outside container is not sufficient, resulting in a sink condition violation [15]. In the reverse dialysis method, the drug released from microparticles placed in the external release medium can easily diffuse into the dialysis tubing [56]. The third method is side-by-side dialysis, in which the donor and acceptor cells have the same volume capacity and are separated by a dialysis membrane. The drug released from the microparticles is evaluated by placing the microparticles on the donor cells and performing sampling on the receptor cells [54].

In addition to the experimental setup and agitation conditions, the MWCO of the dialysis membrane and the ratio between the internal and external release medium volumes are the main parameters for the successful DM method [15,46]. In particular, the selection of an appropriate MWCO is important for the dialysis membrane. The basic premise of the DM method is that the drug released from the particles diffuses through a semipermeable membrane with appropriately sized pores. Dialysis membranes with sufficiently high MWCO are used for in vitro studies to ensure they are not a limiting factor for drug diffusion [24]. Nonetheless, the selection of MWCO is somewhat subjective because the criteria are not clear. For example, MWCO of 3.5–5 kDa for loperamide [57], 8–14 kDa for risperidone [51], cyclic somatostatin [58] and cefquinome [59], and 100 kDa for beta-sheet peptide [60] have been used. The volume contained in the dialysis bag is much smaller than the external medium. To facilitate drug diffusion, the volume of the inner medium is kept 5–10 times less than the volume of the outer medium, providing the driving force to deliver the drug to the outside and maintaining sink conditions [16]. For example, inner medium volumes reported in the literature range from 1 to 10 mL, while external medium volumes are typically much larger, around 40 to 90 mL [61].

Qu et al. prepared cefquinome-loaded PLGA microspheres for lung targeting and evaluated in vitro cefquinome release by regular dialysis method using a dialysis bag (MWCO 8–14 kDa) [59]. The sealed dialysis bag containing the microspheres was immersed in PBS while stirring in a shaking water bath set at 100 rpm. For sampling, the medium was withdrawn to a volume of 2 mL and replaced with an equal volume of fresh release medium. The microspheres showed initial burst for 1 h and constant release for 36 h.

Chaurasia et al. prepared parenteral risperidone-loaded microspheres with different drug-to-polymer ratios (1:1.5, 1:1.75, and 1:2) and evaluated in vitro release profile by regular dialysis method using a dialysis bag (MWCO 12–14 kDa) [51]. Microspheres were immersed in 50 mL of PBS with continuous magnetic stirring at 100 rpm. For sampling, samples were taken from the dialysis bag at each time point and replaced with fresh medium to maintain sink condition. Microspheres with low porosity showed higher burst release followed by a longer lag phase and reached release plateau after 14 days, whereas microspheres with a highly porous structure showed a lower burst release followed by a shorter lag phase and reached release plateau phase within 14 days.

Zhang et al. prepared paclitaxel-loaded PLGA microspheres by the double-emulsion solvent evaporation method and evaluated in vitro release profile by regular dialysis method using a dialysis bag (MWCO 14 kDa) [62]. For the release study, the microspheres were suspended in 5 mL sodium salicylate/PBS medium in a dialysis membrane, and the dialysis bag was immersed separately in a screw cap tube containing 50 mL of the sodium salicylate/PBS medium. The tubes were placed horizontally in an orbital shaker. For sampling, 2 mL of solution in the tube was collected and the tube was supplemented with 2 mL fresh medium. When comparing smooth microspheres with internal sporadic porosity and rough microspheres with highly porous internal structure, the smooth microspheres exhibited roughly a slow linear release pattern, whereas the rough microspheres showed a faster S-curve release pattern.

Chen et al. investigated the release profile of various size fractions (5, 32, 70 and 130 μm) of gefitinib-loaded microspheres, which were prepared using the oil-in-water solvent evaporation method and then fractionated by wet sieving [42]. Prior to in vitro release study, this study embedded microspheres in methacrylated dextran hydrogels to prevent aggregation of microspheres during the incubation conditions without limiting the release of gefitinib from formulations. For in vitro release study, gefitinib microspheres-loaded methacrylated dextran hydrogels were casted in Slide-A-Lyzer MINI Dialysis Devices (MWCO 2 kDa) and incubation was performed in PBS containing 1% Tween 80 under constant shaking. The size-fractionated microspheres showed significant differences in drug release between small microspheres and larger microspheres. Microspheres smaller than 50 μm showed rapid diffusion-based release that reached completion within one week. However, the larger microspheres showed a pattern of sigmoid release that lasted for three months, where diffusion (early stage) and erosion (late stage) dominated drug release.

Zhang et al. studied drug release behavior from fenretinide-loaded PLGA microspheres by incorporating nonionic surfactants (Brij 35, Brij 98, Tween 20, and Pluronic F127) [63]. The in vitro release test was performed on a mesh bag (nylon material) with 1 μm pore size instead of a dialysis bag due to the interaction of the drug and the dialysis bag. The release medium was PBS containing 0.1% Tween 20. The samples were continuously agitated at a constant speed and the release medium was replaced periodically as needed to maintain the sink condition. At predetermined time points, each mesh bag was taken out, lyophilized, and analyzed for drug remaining in the microspheres by HPLC. Microspheres prepared with Brij 98 exhibited reduced initial burst and sustained release over 28 days. The release profile was dependent on the concentration of Brij 98 with a very significant increase in the release rate, especially when the surfactant level was increased from 10% to 20% *w*/*w*.

### 2.3. Continuous Flow (CF) Method

**Figure 3:**
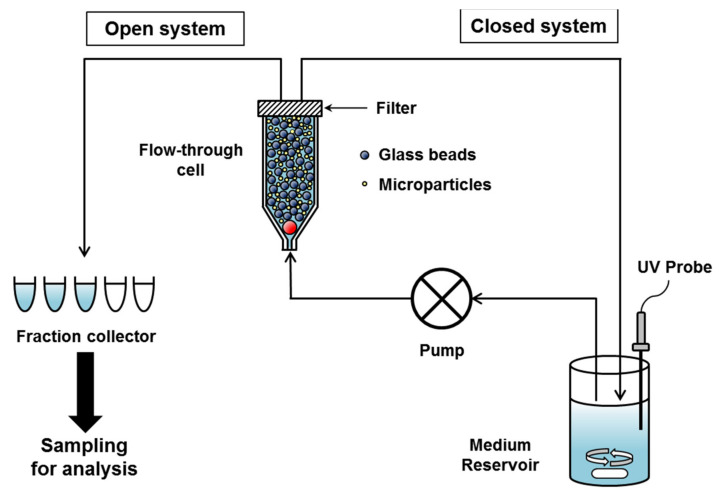
Continuous flow method system for in vitro drug release test of particulate formulations.

The CF method is a drug release testing method using a system equipped with a flow-through cell, a pump and medium reservoir, in which microparticle sample is put into a small volume cell, the release medium flows through the cell by pump, and the released drugs passed through the filter in the flow-through cell are monitored off-line or on-line [64] (Figure 3). In this method, microparticles are separated by filter within the flow-through cell and the released drug can be sampled as often as needed [65]. The CF method attempts to simulate the in vivo environment by continuously flowing a solvent over the immobilized microparticles in order to hydrate the particles and cause dissolution and diffusion of the drug [66]. The limited volume of flow-through cell mimics the injection site of the subcutaneous tissue and the continuous circulating medium around the microparticles mimics the dynamic in vivo environment [67].

For CF method, United States Pharmacopeia (USP) apparatus IV is recommended and many applications for in vitro drug release test of particulate formulations have been reported [67,68,69,70,71,72]. Originally, the USP apparatus IV was developed for in vitro dissolution testing of modified release oral dosage forms, but the diversity of flow-through cell types and the flexibility of medium volume allow it to be applied to a wide range of dosage forms [73,74]. The flow-through cell is mounted vertically with a filter system on the top and has a bottom cone filled with small glass beads (approximately 1 mm in diameter) and one bead (approximately 5 mm in diameter) placed at the apex (Figure 3). Microparticle samples can be placed within a layer of glass beads or mixed with glass beads. Glass beads are useful for preventing aggregation of microparticles, reducing dead volume within cells, and increasing laminar flow [64]. It is also necessary to determine the proper ratio of glass beads to microparticles to avoid backpressure problems [15]. Since the reservoir volume can be adjusted to allow testing of various formulations, the volume can be reduced to measure the concentration of the released drug below the limit of quantitation of the analytical method or increased to facilitate maintaining sink conditions for poorly soluble drugs [66,74].

The amount of drug released can be monitored online by a UV-Vis spectrophotometer or fiber optic probe, or samples can be collected in fractions and analyzed by HPLC or other appropriate method [64,65,69,70,71,72,73]. In particular, the fiber optic UV probe facilitates in situ monitoring of drug release from the microparticles while minimizing detection error due to interference of suspended microparticles and air bubbles caused by stirring [15]. Zolnik et al. have demonstrated the successful application of the USP apparatus IV method in conjunction with fiber optic UV probes to the release testing of dexamethasone-loaded microspheres [64]. This study showed the usefulness of fiber optic UV probes for the acquisition of multiple data points in a short period of time, which enabled a comprehensive characterization of the initial burst release. Voisine et al. showed the application of a fiber optic UV probe in the USP apparatus IV method to simultaneously monitor cefazolin and its degradation products from PLGA microspheres, where absorbance monitoring at the isosbestic point (wavelength where the drug and degradation products have the same absorbance) resulted in approximately 100% release determination for 25 days [65].

USP apparatus IV can be used in open-loop or closed-loop mode (Figure 3). In an open-loop mode, fresh medium from the reservoir continuously passes through the cell and samples are collected in fractions within defined time intervals. The data collected represent the non-cumulative amount of drug released at specific time intervals and the total medium volume used in an open-loop mode can be infinite [73,75]. In a closed-loop mode, a fixed volume of medium is circulated through the cell and the cumulative amount of released drugs can be monitored [64,65]. Since the open-loop mode requires a large amount of medium, the closed-loop mode, which operates with a small amount of medium, is advantageous for microparticles that require long-term testing.

Compared to the SS and DM methods described above, the CF method using USP apparatus IV offers several advantages: (1) continuous and convenient sampling through an automated process, (2) easy maintenance of the sink conditions, (3) minimal aggregation of the microparticles when mixed with glass beads in the flow-through cell, (4) various types of medium with different pH and ionic strength available, (5) flexibility in monitoring drug release through an online detection system, and (6) better reproducibility of results based on a compendial apparatus with well-defined geometry and hydrodynamics [15,16,17,75,76,77]. However, disadvantages of this method can also arise when long-term testing over weeks to months is required. During long-term release test, failure of the O-rings and filters in apparatus components may occur, and small particles can clog the filters, causing variation in the flow rate and back-pressure problem [15,16,17]. In case of protein-loaded microparticles, the slow and incomplete release from microspheres can occur due to adsorption of proteins onto the hydrophobic surfaces of the USP apparatus IV such as glass beads, flow-through cell surface, filter, and tubings [78]. Rawat and Burgess suggested that incorporating surfactants such as sodium dodecyl sulfate (SDS) into the release medium would be useful for accurately estimating the cumulative release of proteins from the microparticles by inhibiting protein adsorption to the hydrophobic surfaces of the apparatus [78].

Recently, Andhariya et al. conducted in vitro release testing of naltrexone–loaded microspheres and a commercial product Vivitrol^®^ by the CF method using USP apparatus IV [79]. In this study, microparticles were mixed with 1 mm glass beads and the release medium (10 mM PBS (pH 7.4) containing 0.02% Tween 20 and 0.02% sodium azide) was circulated through the flow-through cells in a closed-loop mode at a flow rate of 8 mL/min. The release medium in the reservoir was replaced every five days. This study showed that the CF method using the USP apparatus IV has the ability to detect differences in the in vitro release performance between differently manufactured microparticles.

Kohno et al. used USP apparatus IV-based CF method to investigate the in vitro release performance of risperidone-loaded microparticles prepared with PLGAs of different molecular weights [80]. In this study, microparticles were mixed with 1 mm glass beads and the release medium was circulated through the flow-through cells in a closed-loop mode at a flow rate of 8 mL/min at 37 °C. Two different release media was used: (1) 10 mM PBS (pH 7.4) with 0.01% sodium azide and (2) 10 mM HEPES (pH 7.4) with 0.02% sodium azide, 99 mM NaCl and 0.02% Tween 20. The release profiles were similar for both release media, but the release rate was slightly faster in the HEPES buffer. This was considered to be due to the presence of the surfactant Tween 20 in HEPES buffer, which can promote wetting of PLGA microparticles, resulting in faster buffer penetration into the microparticles during release testing [81].

Tipnis et al. studied USP apparatus IV-based CF method for in vitro release testing of triamcinolone acetonide-loaded PLGA microspheres [82]. In this study, microparticles were dispersed with glass beads and the release medium (10 mM PBS (pH 7.2) containing 0.1% SDS and 0.01% sodium azide) was circulated through the flow-through cell in a closed-loop mode with a flow rate of 8 mL/min at 35 °C. This condition was optimized by testing various conditions of release medium-based conditions (ionic strength, surfactant concentration, and medium volume for drug solubility) and instrument-based parameters (flow rate and temperature). Among several parameters, temperature was identified as an important parameter because the rate of drug release at 39 °C was much slower than those at slightly lower temperatures, 35 and 37 °C. As evidenced by the morphology of the interior pores of microparticles, the slow drug release at 39 °C was thought to be due to polymer plasticization, which trapped the drug crystals within the microparticles and hindered pore channeling. Therefore, this study indicates that precise control of temperature can be a critical condition for obtaining an appropriate in vitro release profile of the microparticles.

### 2.4. Characteristics of In Vitro Drug Release Testing Methods

The available methods have their own advantages and disadvantages, so the choice of methods and conditions should be carefully considered. Table 2 summarizes the characteristics of in vitro drug release testing methods for microparticulate formulations. The SS method is easy to perform and can use USP apparatus II as compendial apparatus [15]. This method is often useful for measuring initial burst release, but it suffers from cumbersome sampling process and undesirable withdrawal potential of particles from the medium. The DM method does not require time-consuming procedure to separate particles from samples obtained for analysis of released drug, because the particles and the medium to be sampled are already physically separated by a membrane. Therefore, the DM method minimizes the possibility of particle loss during sampling process. Disadvantage of this method is that due to the inherent barrier properties of the dialysis membrane, the equilibrium between the inside and outside of the dialysis bag is slowed, which may limit the accurate analysis of initial drug levels in formulations with high burst release [45]. Additionally, this method cannot be used if the drug is bound to particles or dialysis membrane. The CF method uses USP apparatus IV and offers several advantages based on an automated process [65,66,67,68,69,70,71,72,73]. Due to the limited number of sampling points, SS and DM methods are labor intensive and less accurate for testing drug release profiles in just seconds or minutes or the first few minutes. The CF method overcomes the limitations of SS and DM methods by providing continuously automated sampling and analysis through online measurements. The online measurements offer advantage of gapless monitoring of drug release, but have the disadvantage of relatively low sensitivity with a higher quantitation limit than conventional analytical methods such as HPLC [46]. Disadvantages of the CF method are that it requires expensive, maintenance-intensive equipment and may have a difficulty in test for long periods of time over weeks to months, due to failure of O-rings and filters in device components [15,16,17].

## 3. Accelerated In Vitro Release Testing Methods

Since drug release from microparticles typically takes weeks to months, real-time in vitro release testing methods are time-consuming and difficult to screen many formulations to optimize final formulation. For microparticles that release drug for more than a month, preservatives need to be added to perform real-time drug release testing at 37 °C, and the stability and compatibility issues of release test device components such as tubings and membranes may arise [16]. Therefore, the development of an accelerated testing method that can shorten the experiment period is attracting attention so that the formulation can be quickly evaluated during development and manufacturing process [15,16,67,83].

Accelerated release test method can be used for quality control purposes by distinguishing microparticle formulations with different release properties in vivo [84,85,86]. Since biopolymeric microparticles are typically characterized by a three phase release profile, the accelerated testing is recommended to be correlated with real-time release using initial time points, intermediate time points, and time points exceeding 80% of the cumulative amount released [68]. Ideally, accelerated test method should be able to evaluate any initial burst release, but if the burst release phase is very fast, it can be difficult to distinguish under accelerated conditions. In such cases, a real-time release study should be performed along with an accelerated release studies to assess the burst release phase [15].

Accelerated drug release can be achieved by altering one or more conditions employed in a real-time in vitro release study. Such conditions include elevated temperature, pH, test medium composition, surfactants, organic solvents, and agitation rate (Table 3) [84,85,86]. In accelerated testing, the drug release mechanism should not be altered, only the release rate should be accelerated [87]. However, extreme conditions that accelerate drug release can lead to changes in the drug release mechanism. Therefore, it is important to investigate and understand how the parameters used in accelerated tests may affect drug release mechanisms [67]. In addition, it should be noted that extreme conditions may affect drug stability and produce degradation products of medium components.

Elevated temperature is the most commonly used parameter for the accelerated drug release test because it can effectively accelerate drug release by increasing the mobility of polymers in the microparticle matrix and enhancing drug diffusion from microparticles [37,88,89,90]. It has been reported that the drug diffusion coefficients can increase by up to three orders of magnitude at temperatures near the glass transition temperature (Tg) of the polymer [91]. Furthermore, high temperature can accelerate erosion-controlled drug release by enhancing the hydration and degradation of polymers. In general, it is recommended that the temperature should not be higher than the Tg of the polymer, as the release mechanism can change at temperatures above the Tg [15]. Plasticization of polymers at high temperature can lead to changes in microparticle morphology (e.g., microparticle surface pore closure and particle aggregation), which may reduce the rate of drug release and especially affect burst release in the initial release phase [92]. Zolnik et al. studied an accelerated drug release method of four different PLGA (MW 5, 25, 28 and 70 kDa) microparticle formulations using USP apparatus IV at elevated temperatures (45, 53, 60, and 70 °C) [90]. At real time (37 °C), microparticles prepared with 5 kDa PLGA exhibited diffusion-controlled kinetics, whereas microparticles prepared with 25, 28 and 70 kDa PLGAs followed erosion-controlled kinetics. The accelerated test was able to predict real-time release for erosion-controlled release microparticles, but it was not suitable for diffusion-controlled release microparticles. All four formulations exhibited morphological changes of microparticles including surface pore closing at elevated temperature with consequent reduction in initial burst release. This study indicates that accelerated release test using elevated temperatures should be reinforced by real-time studies that allow adequate assessment of the initial burst release.

Release test medium conditions to accelerate drug release from polymeric microparticles include pH and organic solvents [93,94,95]. Acidic and basic pH conditions catalyze hydrolysis in the ester backbone of PLGA or PLA, resulting in polymer degradation into shorter chain alcohols and acidic oligomers [96,97]. However, the mechanism of polymer erosion appears different under acidic and basic pH conditions. In acidic conditions, the polymer undergoes bulk erosion similar to the degradation properties obtained at pH 7.4, whereas in basic conditions (pH > 13), degradation follows surface erosion [98]. Polymer degradation leads to accumulation of acidic degradation products within the microparticles, and the acidic microclimate pH can then accelerate polymer degradation, forming channels through in which drug release takes place [99]. Compared with elevated temperatures, pH does not have a significant effect on accelerating drug release and extreme pH conditions may not be suitable for drugs that are not stable under these extreme pH conditions [67]

Organic solvents such as acetonitrile and ethanol have been used to accelerate drug release from polymeric formulations [83,100,101,102]. Kamberi et al. demonstrated that adding acetonitrile to the release medium can accelerate drug release by increasing the porosity of the PLGA-based drug-eluting stent matrix [83]. This method was used to optimize experimental variables in the manufacturing process and showed good correlation with real-time release at 37 °C. Xie et al. performed accelerated release study of thymopentin from PLGA microspheres by adding ethanol to the release medium at a concentration of 20% (*v/v*) [101]. In addition to the medium containing 20% ethanol, this study used a gradient heating program consisting of 3 stages and each stage with different temperatures for the correlation between short-term release and real-time release.

Here are some of the recently published research papers related to accelerated release tests.

Tomic et al. studied the effects of several parameters (pH, osmolarity, ionic strength, and temperature) on accelerated release of peptide (cyclic somatostatin analog)-loaded PLGA microspheres [58]. This study recommended the CF method using USP apparatus 4 under conditions of 0.02 M PBS at pH 2 and 45 °C. Changes in pH (4 to 2) and temperature (40 to 45 °C) increased the rate of peptide release without significant changes in the release mechanism. On the other hand, when the buffer concentration was decreased from 0.02 M to 0.01 M, the release rate of the peptide increased and the release mechanism was changed from tri-phasic to bi-phasic, while ionic strength did not have any effect on peptide release.

Shen et al. studied accelerated release testing methods of risperidone-loaded microparticles with different internal structure and porosity [103]. In this study, SS and CF methods were tested using elevated temperature (45 °C). Both the SS and CF methods showed the ability to distinguish formulations with different porosities, but for risperidone microparticles with high porosity, only the CF method using USP apparatus 4 showed a good reproducibility under accelerated test conditions at 45 °C. The low reproducibility of the SS method for highly porous microparticles appeared to occur by the inconsistent sampling process due to the flotation of the microparticles in the release medium.

Garner et al. also studied accelerated release testing methods of risperidone-loaded microparticles, but in this study they used the SS method using an orbital agitation incubator [104]. To optimize the accelerated release conditions, the effects of vessel type (centrifuge tube, glass tube, glass flask, and glass jar), sampling volume, solid beads, and agitation speed on risperidone release were investigated. Significant particulate aggregation was observed in narrow diameter vessels such as centrifugal tubes and glass tubes, resulting in a slower release rate, whereas minimal particulate aggregation was observed in wide diameter vessels such as glass vessels and glass flasks, resulting in higher release rates. In the sampling volume study, a sampling volume of 1 mL (out of 40 mL total) showed a longer lag phase compared to a 30-mL sampling volume. This difference could be attributed to minimized particle aggregation due to additional agitation and violation of the sink condition. This study demonstrated that the SS method using orbital agitation is a simple, cost-effective and reliable method for testing the release properties of risperidone-loaded microparticles.

## 4. In Vitro–In Vivo Correlation (IVIVC)

In vivo drug release testing using animal models is desirable for characterizing the performance of microparticles, but it is time-consuming, expensive, and labor-intensive to plan and perform. On the other hand, in vitro drug release testing, a surrogate for in vivo studies, is much simpler and less expensive to perform, and can be done in a short time through the accelerated release test method. Therefore, In vivo measurements of drug release from injectable formulations are desirable, but they are time consuming, expensive and labor intensive to plan and perform. IVIVC study between in vitro drug release and in vivo bioavailability is increasingly becoming an integral part of microparticle product development [18,19,20,21,22,23].

**Figure 4:**
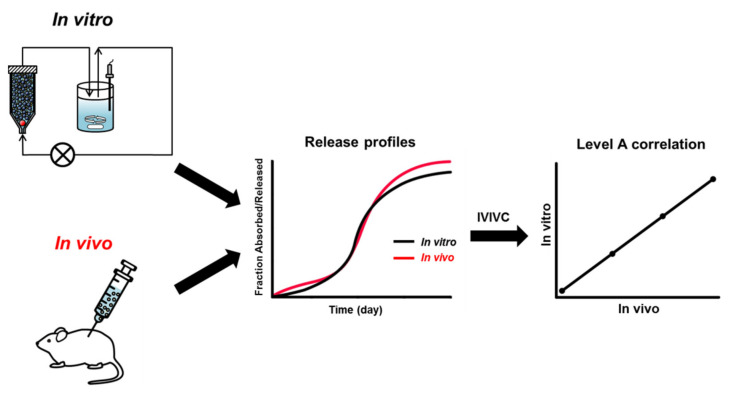
Schematic representation of Level A correlation between in vitro release profiles and in vivo release profiles of particulate formulations.

According to the US FDA guidance published in 1997, IVIVC is defined as “a predictive mathematical model describing the relationship between an in vitro property of an extended release dosage form and a relevant in vivo response” [105]. The US FDA guidance provides the levels of correlation: Levels A, B, C, D, and multiple-level C. Level A, the highest correlation, represents a point-to-point relationship between in vitro dissolution and in vivo absorption over time. With a Level A IVIVC, the in vitro drug release profiles are directly superimposable with in vivo absorption curves or can be made to be superimposed using an appropriate scaling factor (Figure 4). Level B is the correlation between summary parameters such as in vitro dissolution rate and in vivo absorption rate, for example, mean dissolution time (MDT) vs. mean residence time (MRT). Level C is a single point relationship between a dissolution parameter (e.g., amount dissolved at a particular time) and in vivo pharmacokinetic parameters (e.g., the peak plasma concentration (C_max_) or area under the curve (AUC)). Thus, the Level C correlation does not describe the complete shape of the in vivo release profile. Multiple-level C is a correlation developed for many points over the entire release profile by comparing multiple in vitro dissolution time points with one or more in vivo pharmacokinetic parameters. Level D is a rank order correlation that is qualitative. Of these IVIVC levels, the Level A is considered to be the most informative and the only IVIVC level available to obtain a biowaiver [21,22,23].

The setting of meaningful IVIVC is useful to guide formulation and process changes at various stages of drug product development. In addition, IVIVC can be used to support and validate the use of in vitro drug dissolution methods and can help establish clinically relevant in vitro release specifications. Most importantly, once the correlation between drug release in vitro and in vivo is established and validated, the in vitro drug release method can be used as a surrogate for bioequivalence studies [15]. Through the successful development and application of meaningful IVIVC, the in vivo drug performance can be accurately predicted from the in vitro performance of drug products. Thus, establishing meaningful IVIVC minimizes the need for human or animal studies [19,20,21,22,23].

**Figure 5:**
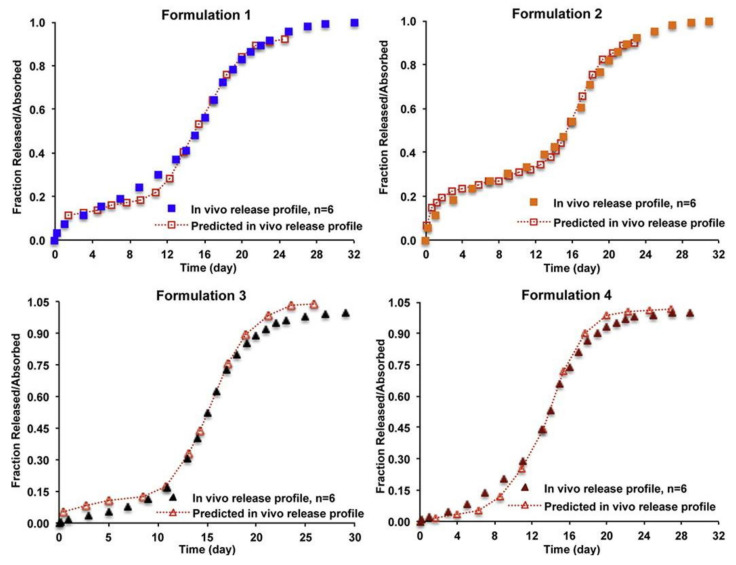
Predicted in vivo release profiles of different risperidone microparticle formulations based on established Level A IVIVCs and the overlapping with their experimental profiles. Reprinted with permission from ref. [81]. Copyright 2015 Elsevier.

There are a few publications on IVIVC of PLGA microparticles as summarized in Table 4. Among them, the most research papers on risperidone microspheres have been published. D’Souza et al. reported a Level A IVIVC of risperidone-loaded PLGA microspheres prepared two copolymers of PLGA 50:50 and PLGA 75:25 [106]. In vitro drug release study was performed using the DM method at 37 °C. In vivo release profiles were obtained through deconvolution method using the Nelson–Wagner equation and fractional AUC approach. A good linear correlation between drug release in vitro and the amount of drug absorbed in vivo was confirmed by an almost 1:1 correlation (*R*^2^ values > 0.97) between in vitro release and in vivo performance. Shen et al. investigated IVIVC of risperidone microspheres made by different manufacturing processes with the same formulation composition [81]. In vitro release was conducted using two different methods, SS and CF using USP apparatus 4. In vivo pharmacokinetic profiles following intramuscular administration were determined using a rabbit model. The pharmacokinetic profiles were deconvoluted using the Loo–Riegelman method and Level A IVIVCs were established based on in vitro release data obtained with the CF method. The developed IVIVCs were used to predict the in vivo profile of the microparticle formulations not used for IVIVC development, where the predicted in vivo release profiles almost overlapped with their experimental in vivo release profiles (Figure 5). Hu et al. examined the relationship between in vitro risperidone release from PLGA microsparticles under accelerated release condition and in vivo absorption in rats [102]. In vitro release study was performed using the SS method in the 0.1 M PBS (pH 7.0) with 20% ethanol at 45 °C. In vivo study was carried out by subcutaneous administration of risperidone microparticles at dose of 40 mg/kg in rats. IVIVC of two formulations of risperidone microparticles was established by Wagner-Nelson model, Loo-Riegelman model, and numerical deconvolution model. The *R*^2^ values > 0.97 were obtained in Wagner–Nelson model and numerical deconvolution model. This study shows that the accelerated release method can be useful for predicting the in vivo drug absorption of risperidone-loaded microparticles.

Andhariya et al. studied the effect of variable burst release on the predictability of IVIVCs of microparticles [107]. In this study, two microparticles, Risperdal Consta^®^ (risperidone) and Lupron Depot^®^ (leuprolide acetate), were investigated. In vitro release study of risperidone-loaded microparticles was performed using the CF method with USP apparatus 4, while in vitro release study of leuprolide acetate-loaded microparticles was carried out by the SS method in the 33 mM PBS (pH 7.4) containing 0.02% Tween 20 and 0.02% sodium azide at 37 °C. In vivo study was conducted using rabbit model by intramuscular injection of microparticles. IVIVC was assessed using a two-stage based deconvolution approach with time scaling and shifting factors. This study showed that the development of IVIVC using microparticles formulations with low variable burst release significantly improved the ability to accurately predict drug release properties, whereas microparticles formulations with highly variable burst release impaired IVIVC’s ability to predict the in vivo release properties of microparticles. Therefore, IVIVC developed using the low burst release was difficult to predict formulations with high burst release and vice versa. Thus, this study provided a comprehensive understanding of the impact of the variable burst release phase on the development of IVIVC for microparticles. Previously, it has been reported that the high burst release observed under in vitro studies can be obscured by the in vivo absorption phase at the intramuscular injection site, fibrous encapsulation of microparticles through the host immune response or steric hindrance by the extracellular matrix [22,69,108,109].

As other examples of IVIVCs of microparticle formulations, D’Souza et al. showed nearly 1:1 linear Level A correlation between in vitro release (DM method) and in vivo release in rats of olanzapine-loaded PLGA microparticles [19], Andhariya et al. has demonstrated that a Level A IVIVC between in vitro release (CF method using USP apparatus IV) and in vivo release in rabbits of naltrexone-loaded PLGA microparticles [79], and Guo et al. reported a good correlation between in vitro release (SS method) and in vivo release in rats for donepezil-loaded PLGA microparticles [38]. Recently, Park et al. reported good IVIVC of PLGA microparticles containing norquetiapine (*N*-desalkyl quetiapine), which is an active metabolite of quetiapine, where point-to-point relationship of *r*^2^ greater than 0.98 between in vitro release (SS method) and in vivo release in rats was obtained [110]. Recently, Kaihara et al. developed an in vitro release testing method by rotating a customized paddle inside a dialysis membrane to predict the in vivo drug release properties of tacrolimus-loaded microparticles [111]. This study found that it may not be possible to predict both the overall drug release profile and the initial burst using a single method. The developed paddle method using a dialysis membrane was useful for predicting the overall pharmacokinetic profile of tacrolimus-loaded microparticles, but not for correlation with initial burst within 1 day. For the initial burst correlation, the conventional paddle method showed better performance. This study proposed a combination of a conventional paddle method and a novel paddle method (including dialysis membrane and internal agitation) to predict both the initial burst and overall drug release profile of microparticle formulations.

## 5. Stability of Drugs in PLGA Particulate Formulations

There have been many reports on stability issue of peptides and proteins in the PLGA-based particulate systems [112,113,114,115,116,117]. Drug stability issues in PLGA formulations can arise not only during manufacturing and storage, but also during drug release process. Drugs in microparticles can be subjected to unfavorable microenvironments created by the degradation of PLGA polymers during drug release period. PLGA is degraded by hydrolysis of ester bonds in the polymer backbone and the degradation products (lactic acid, glycolic acid, and their oligomers) accumulate inside the particles, resulting in acidic microenvironment with the minimum pH as low as 1.5 [118,119,120]. This acidic microenvironment inside the particles can trigger several physicochemical degradation reactions, e.g., acylation, deamidation, amide bond hydrolysis, protein denaturation and aggregation, and the drug instability remains as one of the major obstacles in the development of PLGA depot formulations [121,122].

**Figure 6:**
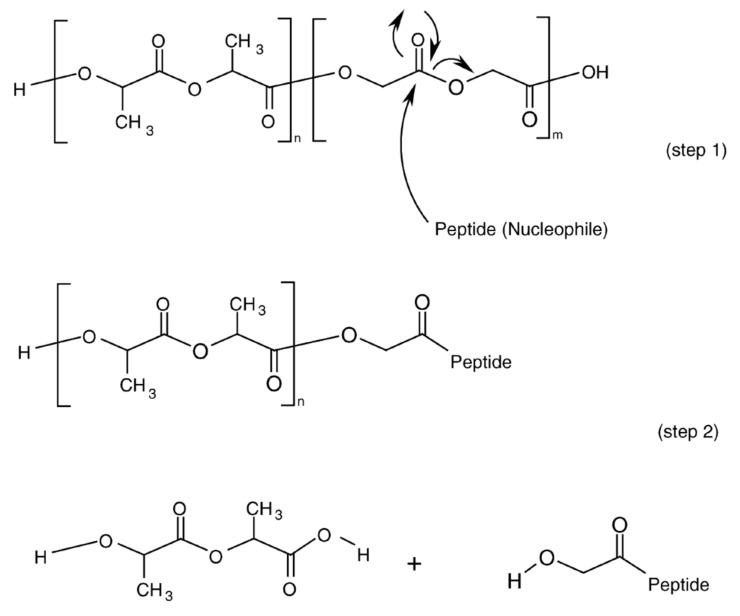
Proposed mechanism of acylation reaction of peptides incorporated into PLGA microparticles. Reprinted with permission from ref. [36]. Copyright 2005 Elsevier.

Acylation of peptides incorporated into PLGA microparticles has been shown to account for a significant portion of the drug instability problem in drug release process of PLGA microparticles [122,123,124,125,126,127,128,129]. Nucleophilic groups of peptides, mainly the primary amines of the N-terminus or Lys residue, are the major targets for peptide acylation [36,124,130] (Figure 6) and the peptide acylation can affect the release properties, biological activity, pharmacological effect, and toxicity of peptide drugs in PLGA microparticles [131,132,133]. In addition to primary amines, arginine residue has also been identified as acylation site in goserelin and leuprolide having no primary amines [128,129]. Several strategies have been proposed and studied to prevent and minimize peptide acylation in PLGA formulations [134,135,136,137,138,139,140,141,142,143,144,145].

Recently, Li et al. characterized the degradation products of exenatide, such deamidation, oxidation, and acylation products, during in vitro release evaluation of exenatide-loaded PLGA microparticles [122]. The in vitro release study was performed by the SS method in the 10 mM PBS (pH 7.4) containing 0.02% Tween 80 and 0.02% sodium azide at 37 °C under mild agitation. Peptide acylation was found to be the most prominent degradation reaction during in vitro release, with acylated peptides steadily increasing during release compared to the parent peptide, making it the most abundant peptide species released from microparticles in the late phase of release. The multiple primary amines of exenatide in N-terminus and two Lys residues (Lys-12 and Lys-27) can react with the ester backbone of PLGA to form acylated peptides [127,132,141].

In peptide-loaded PLGA/PLA microparticles, the presence of acylated peptides is an essential element to be analyzed during in vitro release test period. For this, an analytical method capable of distinguishing between intact and acylated peptides must be established. In some cases, it may be necessary to further confirm the biological activity and toxicity of the acylated peptides, including immunogenicity [131,132,133].

## 6. Conclusion and Future Perspectives

In this review, the in vitro drug release testing of biopolymeric microparticles was categorized into SS, DM, and CF methods, and their characteristics were summarized with the recent studies. Each method has its own advantages and disadvantages (Table 2), so the method and conditions should be carefully selected and optimized. In the research phase, the SS or DM method is useful because they are easy to perform and can process many samples simultaneously, while CF method requires expensive equipment, and the number of flow-through cells in USP IV apparatus is usually fixed at 7, making it difficult to process more than seven samples at a time. The CF method is best suited for quality control due to its automated process and excellent reproducibility.

Accelerated in vitro drug release testing is attractive because it can shorten the evaluation time of long-term release formulations during development and manufacturing process. For this purpose, the accelerated release testing must produce drug release profiles that correlate well with those obtained from real-time in vitro drug release testing. Elevated temperature (typically 45 to 70 °C) is the most commonly used method for accelerated drug release testing [90], in which Tg of the polymer and microparticle morphology changes due to plasticization of polymers need to be considered [15,92]. The accelerated in vitro drug release testing methods can be useful for establishing IVIVC of microparticle formulations [102]. IVIVC is becoming an essential part of long-term release microparticle formulations because it may allow predicting drug performance in vivo and thus minimize the need for human or animal studies. Recently, Level A IVIVCs have been established for several microparticle products (Table 4), but meaningful IVIVC development still remains a challenge because of the complex nature of microparticles that typically show multiphasic release profiles and the lack of a compendial or biorelevant in vitro drug release testing methods [21]. Therefore, more efforts are needed in research to develop a universally usable standardized in vitro release testing method to achieve accurate and reproducibility data.

Microparticle-based products such as Lupron Depot and Sandostatin LAR have been on the market since 1996 and 1997, respectively (Table 1), but to date, no generic products have been approved by the FDA. A generic product must be qualitatively (Q1) and quantitatively (Q2) the same as the reference-listed drug (RLD) products [146]. Among the parameters affecting the release profiles of drug from microparticles, the properties and content of polymers and other inactive ingredients used in generic product can well meet the requirements for Q1/Q2 sameness. However, if manufacturing methods are different, Q1/Q2 sameness may not guarantee the same drug release kinetics and bioavailability between generic and RLD products [81,104]. Besides, minor changes in manufacturing processes can also affect in vitro/in vivo performance of Q1/Q2 equivalent microparticles [71,147,148]. Robust and reproducible in vitro release testing methods can be useful to ensure consistent product performance, and IVIVC can play an important role in generic product development. Level A IVIVC is preferred to use in vitro drug release data as a surrogate for bioequivalence among the levels provided by FDA guidance [105]. Consequently, the development of biorelevant and reliable in vitro release testing method is required for effective quality control of long-term release particulate formulations and generic product development.

## Figures and Tables

**Table 1:** PLA/PLGA-based microparticle formulation products approved by EMA and FDA.

Product	Drug	Company	Administration Route	Indications (Approval Year)
Arestin	Minocycline hydrochloride	OraPharma	Periodontal	Adult periodontitis (2001)
Bydureon	Exenatide	Amylin	SC	Type 2 diabetes (2012)
Decapeptyl	Triptorelin acetate	Debiopharm	IM	Prostate cancer (1986)
Lupron Depot	Leuprolide acetate	TAP Pharmaceuticals	IM	Prostate cancer (1996)
Lupron Depot-PED	Leuprolide acetate	TAP Pharmaceuticals	IM	Central precocious puberty (2011)
Risperdal Consta	Risperidone	Janssen Pharmaceuticals	IM	Schizophrenia (2003)
Salvacyl LP	Triptorelin acetate/embonate	Ipsen	IM	Severe sexual deviations (2006)
Sandostatin LAR	Octreotide acetate	Novartis	SC	Acromegaly (1997)
Somatuline Depot	Lanreotide acetate	Ipsen-Beafour	SC	Acromegaly (2007)
Suprecur MP	Buserelin acetate	Sanofi-Aventis	IM	Endometriosis (2002)
Trelstar Depot	Triptorelin pamoate	Debiopharm	IM	Prostate cancer (2000)
Triptodur	Triptorelin pamoate/embonate	Debiopharm	IM	Central precocious puberty (2017)
Vivitrol	Naltrexone	Alkermes	IM	Alcohol dependence (2006), Opioid dependence (2010)
Signifor LAR	Pasireotide pamoate	Novartis	IM	Acromegaly (2014)
Zilretta	Triamcinolone acetonide	Flexion Therapeutics	IA	Osteoarthritis pain of knee (2017)

EMA, European Medicines Agency; FDA, Food and Drug Administration; IA, Intra-articular; IM, Intramuscular; PLA, poly(lactide) (PLA); PLGA, poly(lactide-co-glycolide); SC, Subcutaneous.

**Table 2:** Major characteristics of in vitro release testing methods for polymeric particulate formulations.

Methods	Sample and Separate (SS)	Dialysis Membrane (DM)	Continuous Flow (CF)
Compendial apparatus	USP apparatus II (paddle)	-	USP apparatus IV (Flow-through cell)
Sample container	Tube or Basket	Dialysis sac	Flow-through cell
Sampling	Sampling supernatant of the release medium after particle separation by centrifugation or filtration	Sampling bulk media outside the dialysis sac containing the microparticles	Sampling from a reservoir where the release medium is circulated through the cells containing the microparticles
Advantages	Easy to perform and accurate measurement of the initial burst drug release	Convenient sampling and minimal particle loss	Automated process, multiple time points sampling available and in situ detection methods applicable
Disadvantages	Cumbersome sampling process and undesirable withdrawal of microparticles from the medium	Slow equilibration with the outer media leading to inaccurate measurement of initial drug levels	Expensive and maintenance-intensive equipment, and the difficulty of long-term release testing

**Table 3:** Summary of factors and in vitro release methods studied for accelerated in vitro release testing of polymeric microparticle formulations.

Factor	Condition	In Vitro Release Method	Drug	Reference
Temperature	45 °C	CF	Risperidone	[87]
	40, 50, 55, 60 °C	SS	Leuprolide	[37]
	50, 55, 60 °C	DM	Leuprolide	[89]
	45, 53, 60, 70 °C	CF	Dexamethasone	[90]
	45, 53, 60, 65 °C	DM	5-fluorouracil	[94]
	40, 45, 50, 55 °C	SS	Thymopentin	[101]
	45, 50, 55 °C	SS	Risperidone	[102]
pH	2.4	CF	Dexamethasone	[93]
	1.3, 7.4, 10.8	DM	5-fluorouracil	[94]
	4.7, 7.0	SS	Thymopentin	[101]
	5.0, 7.0, 9.0	SS	Risperidone	[102]
Organic solvent	Acetonitrile, ethanol, acetone 10% (*v*/*v*)	SS	Thymopentin	[101]
	Ethanol 10, 20, 30% (*v*/*v*)	SS	Risperidone	[102]
Osmolarity	280, 370, 560, 700, 840 mOsm/L	DM	5-fluorouracil	[94]
	300, 500, 700 mOsm/L	SS	Risperidone	[102]

CF, Continuous flow; DM, Dialysis membrane; SS, Sample and separate.

**Table 4:** Examples of Level A IVIVC of microparticulate formulations in animal models.

Drug Encapsulated in Microparticles	In Vitro Methods	In Vivo Animals	*R* ^2^	References
Donepezil	SS	Rats	>0.97	[38]
Leuprolide	SS	Rabbits	>0.97	[22]
Naltrexone	CF	Rabbits	>0.94	[79]
Norquetiapine	SS	Rats	>0.98	[110]
Olanzapine	DM	Rats	>0.96	[19]
Risperidone	SS	Rats	>0.95	[102]
	DM	Rats	>0.97	[106]
	CF	Rabbits	>0.97	[81]

CF, Continuous flow method; DM, dialysis membrane method; IVIVC, in vitro–in vivo correlation; *R*^2^, the square of the correlation coefficient; SS, sample and separate method.
